# Predicting the sport level in elite male motorcycle speedway riders based on physical profile and experience

**DOI:** 10.1371/journal.pone.0324853

**Published:** 2025-05-22

**Authors:** Stefan Szczepan, Zofia Wróblewska, Faye Perkins, Maciej Markowski, Kamil Michalik

**Affiliations:** 1 Department of Swimming, Faculty of Physical Education and Sport, Wroclaw University of Health and Sport Sciences, Wroclaw, Poland; 2 Faculty of Pure and Applied Mathematics, Wroclaw University of Science and Technology, Wroclaw, Poland; 3 Health & Human Performance, Emerita Professor, University of Wisconsin-River Falls, River Falls, Wisconsin, United States of America; 4 Faculty of Physical Education and Sport, Wroclaw University of Health and Sport Sciences, Wroclaw, Poland; 5 Department of Human Motor Skills, Faculty of Physical Education and Sports, Wroclaw University of Health and Sport Sciences, Wroclaw, Poland; Kasetsart University Faculty of Veterinary Medicine Kamphaengsaen Campus, THAILAND

## Abstract

The main objective of this research was to analyze the physical profile and experience of elite male motorcycle speedway riders, in order to develop a mathematical model to predict their level of sport performance. One hundred and eleven male professional motorcycle speedway riders in the top Polish motorcycle speedway league participated in this study (age: 25.04 ± 6.03 years, height: 172.28 ± 4.76 cm, weight: 65.60 ± 4.66 kg). The riders were divided by the k-means clustering method into three groups of sport level: high (n_1_ = 31), medium (n_2_ = 30), low (n_3_ = 50). To define sport level, several parameters were normalized and aggregated into a new conceptual metric of sport level called ‘Indicator of Sport Level’ (ISL). Body composition assessment, Wingate Anaerobic Test on a cycloergometer, and analysis of acute cardiorespiratory and biochemical responses were performed. One-way ANOVA tests were performed for independent samples, and Pearson’s r linear correlation was calculated between clusters of riders. Multiple linear regression (MLR) was performed to predict the ISL. The elite male motorcycle speedway athletes were characterized by average age (25.10 ± 5.80 years) and years of experience (9.16 ± 5.62 years), which made them different from other clusters (p < 0.05). Also, they were 1.99% taller (173.02 ± 4.91 cm) than low sport level riders (p = 0.003). The strongest predictive variable of sport level in the regression model (R^2^ = 0.28) was body height (p = 0.043) and sport experience (p < 0.001). The results of this study present the optimal range of physical attributes, body height, sports experience, age, and body surface area of the best motorcycle speedway riders and indicate that body height and experience are the most predictive parameters in the model to estimate the sport level of riders.

## Introduction

The sport of motorcycle speedway racing is defined and differentiated from other motorcycle sports by racing on loose-surfaced oval circuits 300–400 m in length including air-fence, starting gates, two corners, the anti clockwise direction of riding, and the broadsiding technique of sliding into bends [1]. Lifting the start tape is the starting signal and riders move as quickly as possible to accelerate to a speed of over 100 kilometers per hour [2]. The other defining aspects of the speedway are the motorcycles have one gear and no brakes, with a maximum engine capacity of 500 cm^3^, and the motorcycle weighing a minimum of 77 kg (allowed from 16 years of age) [3].

A motorcycle speedway match consists of two teams of seven riders who compete in 15 heats [4]. Each match lasts about two hours. In a single heat, four riders (two from each team) complete four laps around the track, which takes about 60 seconds. The aim of each heat is to reach the finish line in the quickest time. The method of awarding points is consistent; 3 points are awarded for winning the heat, 2 points for 2nd place, 1 point for 3rd place, and no points for 4th place. The points gained by each team member, in each heat, are totaled. The team with the most points, wins the match [5]. There are different approaches to assess athletes’ sports level using various diagnostic parameters including a rider’s average heat score (AHS) or average match score (AMS) (the total number of points scored divided by the number of matches) [6].

In Poland alone, three divisions give rise to over 150 annual racing events attracting fans across the age spectrum. The speedway competitive season lasts six months from April until the end of September. Since 2022, eight teams compete in the highest division of Polska Grupa Energetyczna (PGE) Speedway Ekstraliga. The competitive season is divided into the match and rematch phases (14 matches in total). Based upon the top performances during the competitive season, the knockout phase begins where the top four teams (based upon total points during the match and rematch phases) compete in the semi-finals. The two teams that win the semi-finals advance to the final championship event [7].

Motorcycle speedway racing is a multidimensional sport in which a race performance is determined by the anthropometric and physiological aspect of athletes combined with the riders’ interpretation of the tactical situations and environmental demands, such as the mechanical fitting of the bike to the surface, characteristics of the track, and weather [8]. The riders perform relatively short-duration efforts during a single heat lasting about 60 seconds, with a recovery period from two to eight minutes between each heat. During the match, each athlete competes a maximum of 7 times. The physical effort during racing has high demands on the athletes. Recent track testing on the physical demands of riders highlighted the high acute responses (%HR_max_) during every heat [9]. To date, only a limited number of studies have assessed key performance indicators of motorcycle speedway riders that predict their sports level. Martin et al. [10], compared the physical characteristics of high-performing and low-performing motorcycle speedway riders. The findings of this study highlighted shoulder mobility, functional movement, dynamic stability, isometric strength, and anthropometric variables as essential attributes of an elite rider. Michalik et al. [11] investigated the relationship among anaerobic capacity, body composition, acute cardiorespiratory responses in a maximal anaerobic effort, and sport level between male junior and senior speedway riders. Moreover, these authors [11] reported that body height, lean body mass, and body surface area have been shown to be crucial for the high sports level of senior motorcycle speedway riders. It is interesting that another study by Michalik et al. [12] indicated that anaerobic performance, body composition, and cardiorespiratory and biochemical responses in elite motorcycle speedway riders did not significantly change from pre-season measurements to immediately following the competitive season. These results may indicate that these parameters had reached their maximum before the competitive season started and did not improve during the competitive season.

The examination of the physical profile of elite athletes and comparing them with less experienced riders may help understand the main physiological and anthropometric characteristics riders should possess to achieve peak results [13]. Furthermore, knowledge of anthropometrics and physiology of the athletes helps to master the movement techniques, schedule motor function-specific elements of training (apart from the mechanical aspects of bikes), shorten training time and optimize the selection of future talents for motorcycle speedway racing. Previously, a different approach was used (clustering, regression) to predict sport performance [14,15]. For example, to discriminate athletes based on different factors a clustering method has been used [16,17]. In turn, to improve the optimization of an athlete-selection process and predict competitive performance a regression analysis has been performed [15]. The specific sport level was also determined based on experience, anthropometric or physiological variables, and other individual features [15,16,17].

There are many methodology approaches, limitations, and inconsistencies in data available on the physical demands of motorcycle speedway riders. This study is the first to analytically investigate the role of physical profile and experience in elite male motorcycle speedway riders. The first objective of the present study was to create a new statistical measure to compare the sport level of each athlete on the same scale. The second objective was to compare body composition components, anaerobic capacity, peak cardiorespiratory responses, and post-exercise changes in blood gasses and lactate concentration in clusters of male elite motorcycle speedway riders based on sport level. The third objective was to determine the relationship between selected physical characteristics and the sport level of the athletes. The fourth objective was to create a mathematical model to predict the sport level of riders. It was hypothesized that riders with the highest sports level would differ significantly in their physical characteristics from riders from lower sports level.

## Materials and methods

### Participants

The study involved 111 male motorcycle speedway riders of different nationalities from several teams of the highest speedway league in Poland, PGE Speedway Ekstraliga. The athletes were characterized by the following parameters: age: 25.04 ± 6.03 years (95% CI: 23.91–26.18), height: 172.28 ± 4.76 cm (95% CI: 171.39–173.18), weight: 65.60 ± 4.66 kg (95% CI: 64.73–66.48), sport experience: 9.20 ± 5.95 years (95% CI: 8.08–10.32), BMI: 22.12 ± 1.44 kg ∙ m^-2^ (95% CI: 21.85–22.39). All participants were considered professional athletes, including one multiple Senior World Champion, several Senior World Championship medalists, multiple Junior World Champions, Speedway Grand Prix participants, national champions, and national representatives. The following inclusion criteria were used in the study: (a) contracted with a club competing in the PGE Speedway Ekstraliga, (b) at least one competitive season of experience in speedway competitions, (c) age over 16 years, (d) no absolute contraindication to exercise testing as recommended by the AHA guidelines [18]. Athletes were excluded if they sustained an injury three months before the beginning of the study, however, there were no exclusions. Participants took part in the study voluntarily. They were informed about the potential risks of the experiment. All athletes gave their written, informed consent to participate. Athletes were also informed that they could withdraw from the study at any time. None of the participants withdrew from the study. This study was approved by Wroclaw University of Health and Sport Sciences Research Ethics Committee (#10/2015, #21/2022) and conducted in accordance with the Declaration of Helsinki at the Exercise Testing Laboratory (certified PN - EN ISO 9001:2009).

### Study design

The research was conducted between 2015 and 2022, which classifies the research as a retrospective study. All laboratory sessions were conducted in the spring two weeks prior to the start of the competitive season. Research participants reported to the laboratory once to have their blood pressure measured, blood drawn to determine hematological parameters, anthropometric parameters measured, and anaerobic capacity assessed with the anaerobic Wingate test, accompanied by analysis of expiratory gases and heart rate. All laboratory sessions were conducted by the same laboratory technicians and at the same time of day (8:00 AM - 10:00 AM). Firstly, a blood sample was collected between 8:00 and 8:30 AM, after an overnight fast. Secondly, a blood pressure measurement was performed after 10-minutes of sitting. Next, a body composition analysis was conducted. Lastly, all participants performed the Wingate test with cardio-|respiratory and hemodynamic responses analysis. The participants were asked to abstain from heavy exercise, alcohol and caffeine for 24-hours preceding the lab visit.

### Training activity before the competition season

Interviews with head coaches of motorcycle speedway clubs revealed that in the period before the start of the racing season (January – March) riders had their own training protocols to improve their motor skills. The training sessions were created by individual trainers specializing in the development of motor skills based on the motorcycle speedway training methodology approved by the Polish Motor Sports Association [19]. The main training sessions at the gym, focused on strength training (45 minutes/2 times a week) and motor coordination (45 minutes/2 times a week). Endurance exercises consisted of continuous running of several kilometers (45 minutes/twice a week) and cycling (45 minutes/twice a week). Additional training with elements of martial arts, such as kickboxing/boxing (45 minutes/2 times a week). All riders also included exercises stimulating the nervous system and improving the reaction time to the starting signal (table tennis, exercises with tennis balls in pairs) (3 x 40 seconds/1 x week). Specialized track training was complemented by motocross or enduro riding (2 hours/2 x per week). In total, the athlete performed about 13 training sessions per week. Verbal confirmation was received from the head coaches that the presented training protocol was followed by all riders before the investigation was conducted.

### Hematological parameters measurement

Capillary blood was collected at rest from the fingertip before the exercise test to determine morphotic parameters: haemoglobin (Hb) and haematocrit (Ht) concentrations using an ABX Micros OT.16 (Horiba Medical, Kyoto, Japan).

### Anthropometry and body composition measurement

Body height and body weight were measured using a WPT 200 medical scale (Radwag, Radom, Poland). Body composition was determined with a FUTREX analyzer 6100/XL (Futrex Tech, Inc., Gaithersburg, USA) based on the near-infrared spectrophotometry (NIRS) method. The probe was placed on the middle of the bicep’s brachii muscle on the dominant limb. All anthropometry and body composition measurements were performed before the exercise test. The NIRS method has been approved to determine the %FAT in humans [20]. Total body fat percentage (%FM), fat mass (FM) and lean body mass (LBM) were assessed. Body surface area (BSA) was estimated by applying height and weight using the equation for men [21]: BSA = 79.8106 ∙ Height^0.7271 ^∙ Weight^0.398^.

### Anaerobic capacity measurement

To assess anaerobic capacity, the Wingate Anaerobic Test (WAnT) was performed [22]. The test was conducted on an Ergomedic E894 cycle-ergometer (Monark, Vansbro, Sweden). The cycle-ergometer was calibrated before the testing began. A warm-up consisted of five minutes cycling with two, five-seconds “all – out” sprints in the third and fifth minute [22]. After the warm-up, the subjects remained seated on the cycle-ergometer for five minutes. The flywheel load (in kilograms) was 7.5% of the subject’s body weight. The effort lasted for thirty seconds, and the responsibility of the participant was to work at the maximum (possible) frequency of rotations to reach maximum power as quickly as possible and to maintain it for as long as possible. The test subjects were motivated by verbal encouragement to perform the test at the highest intensity possible. After the test, the subject remained on the cycle-ergometer for five minutes. The cycle-ergometer was connected to the computer and Multi Cycle Ergometer v.2.3 software (MCE, Wroclaw, Poland). Peak power output (PPO), total work (TW), which were expressed per kilogram of body weight, and LBM were calculated. A fatigue index (FI) was also calculated.

### Cardio-respiratory and hemodynamic responses measurement

Systolic and diastolic blood pressures were measured at rest after being seated for 10-minutes using an aneroid sphygmomanometer (Riester, Jungingen, Germany). During the WAnT, the subjects breathed through a mask, and the exhaled air was analyzed by a Quark b^2^ device (Cosmed, Rome, Italy). The apparatus was calibrated with atmospheric air and a gas mixture of composition: CO_2_–5%, O_2_–16% and N_2_–79% before the measurements began. Recording of respiratory parameters was done breath-by-breath. Peak lung ventilation (VE_peak_), oxygen uptake (VO_2peak_), and carbon dioxide excretion (VCO_2peak_) were measured. To reduce “noise” and improve data interpretation, the results were averaged every 10 seconds and converted to minute values to exclude erroneous breaths due to coughing, sighing, and swallowing. Heart rate (HR) was measured using an RS400 sport-tester (Polar Electro, Helsinki, Finland) during the Wingate test and recorded by Quark b^2^ analyzer software. For HR, a 10-second average was used, and then the highest HR value was expressed as HR_peak_.

### Blood gasometry and lactate concentration measurement

Capillary blood was drawn from the fingertip into heparinized glass capillary tubes at rest before the start of the test and at the third minute after the end of the test to determine acid-base balance (pH), partial pressure of oxygen (pO_2_), partial pressure of carbon dioxide (pCO_2_) and bicarbonate concentration ([HCO_3_^-^]) using a RapidLab 348 analyser (Bayer, Leverkusen, Germany). Hydrogen ion ([H^+^]) concentration was calculated according to the formula: H^+^ = 10^−pH^, derived from blood pH value. Lactate concentration ([La^-^]) was also measured on a photomer (LP 400 Dr. Lange, Düsseldorf, Germany).

### Sport level parameters measurement

The research used rider classification lists published in the public domain of the organizer of the PGE Speedway Ekstraliga [23]. They consisted of the results of the riders, which were obtained during the analyzed competitive season. The result of the data collection process were numerical sets, including the following diagnostic parameters, which indicated the athletes’ sports level:

- Heats sum (HS) – the number of heats in which the rider participated in the competitive season (0-120 N^o^).
- ◦ Wins sum (WS) – the number of heats won by a rider (0-120 N^o^).
- ◦ Points (PTS) – the total number of points scored by a rider in all heats (0-360 pts.).
- ◦ Points sum + Bonus points (PTS+B) – the total number of points (including bonus points) earned by a rider in all heats (0-360 pts.).

Based on the above results, the following parameters were calculated

- ◦ Percentage of wins (%W) – the percentage of heats won by a rider (0-100%).
- ◦ Heat points average (PTS_av_) – the average number of points scored by a rider per heat (0-3 pts.).
- ◦ Heat Points Average + Bonus, (PTS+B_av_) – the average number of points (including bonus points) earned by a rider per heat (0-3 pts.).

Note: a bonus point is awarded to a rider when he crosses the finish line directly behind his teammate and ahead of at least one rival. However, they are not included in the team’s total score. The number of matches depends on the schedule. The PGE Speedway Ekstraliga features eight teams, so the main phase of the competitive season consists of fourteen rounds (a match and a rematch meeting). This is followed by the knockout phase (a semifinal and a final). In each match, the number of heats in which a rider competes, depends on their age category. A junior rider must compete in a minimum of two heats; the minimum number of heats for senior riders is not regulated. All riders may compete a maximum of seven times in a single match. In addition, the last two heats (14th and 15th, called nominated heats) are theoretically started by the riders with the highest points score in the match (although, the right of choice is held by the coach, who can nominate any rider from the line-up) [3].

### Computation of ‘Indicator of Sport Level’ (ISL)

An additional statistical measure of the riders’ sports level was created to illustrate the sports level of each athlete on the same scale, in dimensionless units. For this purpose, all sport level parameters were normalized (scaled). Normalizations were performed with respect to the maximum value (*x*_*max*_) that the selected seven variables can achieve: HS (120 N^o^), WS (120 N^o^), PTS (360 pkt.), PTS + B (360 pts.), W% (100%), PTS_av_ (3 pts.), PTS + B_av_ (3 pts.) [5]. In the normalization process, the original value of a given parameter (*x*) was divided by (*x*_*max*_) to obtain the range [0–1], where 1 meant the highest value. All normalized variables were aggregated (summed) to obtain the range [0–7], where 7 meant the highest value, and expressed in dimensionless/arbitrary unit (N^o^). In this way, a new conceptual parameter of sport level called ‘Indicator of Sport Level’ (ISL) was obtained. The obtained ISL variable was used for further analyses, i.e. Pearson’s r coefficient correlation and multiple linear regression (MLR).

### Statistics analysis

The quantitative investigation involved a 4-dimensional approach (alpha, power, sample size, and effect size) [24], Due to the retrospective character of this study, it was established post-hoc using G*Power 3.1.9.6 software (Franz Faul, Kiel, Germany) [25]. The obtained average value of effect size for comparison differences between means (F-test) was set at (eta-square η^2^) 0.05 (small). The total sample size was 111 participants. The α level was set at 0.05 and the statistical power (1-β) was estimated at 0.07 [26]. Furthermore, the power analysis procedures addressed multiple linear regression [27]. With the above settings and specifying the number of tested predictors of 26 and the obtained total number of predictors of 4, the power (1-β) power was 0.41.

Python 3.9.7 (Python Software Foundation, Wilmington, USA) was used for statistical processing of data. The results are presented as order statistics: arithmetic mean ± standard deviation (± SD), 95% confidence interval (95% CI lower - upper).

The Shapiro-Wilk test was used to assess the normality of the distribution of the studied features (p > 0.05). The homogeneity of variance was assessed using Levene’s test (p > 0.05). For all analyses, the level of p < 0.05 was considered statistically significant.

From all the riders, groups with similar clusters were extracted using the k-means method. The centers of clusters and the distances of objects from the centers of clusters were selected automatically. The elbow method was used to select the optimal number of clusters, which consisted in identifying on the graph the linear point of decreasing SSE (sum of squared errors) or inertia between SSE and the number of clusters [28]. Clustering was performed based on the following seven parameters of the athletes’ sports level: HS, WS, PTS, PTS + B, W%, PTS_av_, PTS + B_a_ and categorized riders into one of the three groups: high, medium or low.

One-way ANOVA with Tukey post hoc multiple comparisons were used to compare differences in the analyzed parameters between the obtained clusters (groups). The comparisons also indicated the effect size (ES) using eta-square (η^2^) (small = 0.01, moderate = 0.13, large = 0.26) [26].

Pearson’s r coefficient correlation was calculated to examine the relationship between all parameters and the new ISL variable. The analysis was performed among all participants (with and without division into clusters). Correlation coefficient thresholds of 0.1, 0.3, 0.5, 0.7, and 0.9 were interpreted as small, moderate, strong, very strong, and extremely strong correlations, respectively [29].

Multiple linear regression (MLR) performed with the ordinary least squares (OLS) method [30] was used to build a mathematical model predicting the sports level represented by ISL based on all measurements of physical variables. Due to the large number of potential explanatory variables, the stepwise forward selection regression method was used, which meant including independent variables (*x*) in the model that were most correlated with the dependent (explained) variable (*y*). The lowest value of the Akaike information criterion (AIC) was used to select the optimal model. Before performing regression analysis, the fundamental assumptions for using linear regression were investigated and were fulfilled for each case in the presented analysis (correlations, independence of observations, collinearity, normal distribution of residuals, and homoscedasticity). The Durbin-Watson statistical test for autocorrelation was also calculated assuming an acceptable range of 1.50–2.50 [31]. The closer the Durbin-Watson coefficient is to 2, the stronger the assumption, and a value above 2 means dependent observations [32]. Two indicators obtained from the linear regression analysis, namely variance inflation factors (VIF) and tolerance (TOL), were employed to detect the potential multicollinearity problem. The VIF > 4 or TOL < 0.25 indicates a multicollinearity problem [33]. The normal distribution of residuals was examined visually using a normal probability Q-Q plot for the standardized residuals and histogram. Equal variance over the values of the independent variable was evaluated by the Breusch-Pagan test (p = 0.581), where p > 0.05 means homoscedasticity. In order to validate the model of regression (predicted) against the other (observed), a scatter plot has been compared [34]. The performance of models was presented with the root-mean-square error (RMSE) [35]. The Bland–Altman plots were used to evaluate the model’s agreement [36]. Results were presented according to the American Medical Association Manual of Style [37].

## Results

### Clustering and differences of variables in groups

As a result of k-means function applied to the dataset according to their sports level, three clusters were found which consisted of: (1) high sports level, (2) medium sports level, and (3) low sports level. All groups differed significantly in terms of sports level parameters (p < 0.05). The order statistics of the sport level indicators of the riders in created clusters are shown in Table 1.

**Table 1:** Order statistics: mean ± standard deviation and 95% confidence interval of the sport level indicators of the riders.

Variables	Clusters: sport level	mean ± SD	95% CI lower - upper
HS (N^o^ 120)	(1) high	91.36 ± 11.28	87.39 - 95.33
	(2) medium	69.26 ± 8.56	66.19 - 72.32
	(3) low	33.48 ± 4.13	32.33 - 34.62
WS (N^o^ 120)	(1) high	32.11 ± 3.96	30.71 - 33.50
	(2) medium	14.43 ± 1.78	13.79 - 15.06
	(3) low	4.48 ± 0.55	4.32 - 4.63
PTS (360 pts.)	(1) high	168.64 ± 20.82	161.31 - 175.97
	(2) medium	98.09 ± 12.12	93.75 - 102.42
	(3) low	34.98 ± 4.32	33.78 - 36.17
PTS + B (360 pts.)	(1) high	184.42 ± 22.77	176.40 - 192.43
	(2) medium	110.76 ± 13.68	105.86 - 115.65
	(3) low	40.50 ± 5.00	39.11 - 41.88
W% (100%)	(1) high	35.31 ± 4.36	33.77 - 36.84
	(2) medium	20.74 ± 2.56	19.82 - 19.82
	(3) low	12.79 ± 1.58	12.53 - 13.40
PTS_avg_ (0–3 pts.)	(1) high	1.85 ± 0.23	1.76 - 1.93
	(2) medium	1.41 ± 0.17	1.34 - 1.47
	(3) low	1.03 ± 0.13	0.99 - 1.06
PTS + B_avg_ (0–3 pts.)	(1) high	2.02 ± 0.25	1.93 - 2.10
	(2) medium	1.59 ± 0.20	1.51 - 1.66
	(3) low	1.19 ± 0.15	1.14 - 1.23
ISL (N^o^ 0–7)	(1) high	2.42 ± 0.27	2.32 - 2.51
	(2) medium	1.15 ± 0.14	1.09 - 1.20
	(3) low	1.78 ± 0.25	1.71 - 1.84

Note: SD - standard deviation, CI - confidence intervals, HS - heats sum, WS - wins sum, PTS - points, PTS + B - points sum + bonus points, %W - percent of wins, PTS_avg_ - heat points average, PTS + B_avg_ - heat points average plus bonus, N^o^ - number, ISL - conceptual ‘Indicator of Sport Level’. Clusters of rider’s sport level: high (n_1_ = 31), medium (n_2_ = 30), low (n_3_ = 50).

The cluster with the highest sports level was characterized by an experience of 9.16 ± 5.62 years and an age of 25.10 ± 5.80years, which were significantly different between all clusters (p < 0.05). Body height in the highest sports level was 173.02 ± 4.91 cm, which was substantially taller (1.99%) from the lowest sport level (p = 0.003). The medium sports level riders were significantly taller than the low sports level riders (p = 0.001). In contrast, the low sports level riders had significantly smaller body surface area than the high (p = 0.026), and medium sports level riders (p = 0.043) (Table 2).

**Table 2:** Order statistics: mean ± standard deviation and 95% confidence interval with repeated-measures ANOVA and Tukey’s HSD post hoc comparisons of anthropometrics variables.

Variables	Clusters: sport level	mean ± SD	95% CI lower - upper	ANOVA			post-hoc comparison p		
F	p	effect size η^**2**^	1 vs 2	1 vs 3	2 vs 3
Experience (years)	(1) high	9.16 ± 5.62	7.56 - 10.76	2.84	<0.001*	0.05	0.004*	0.004*	0.001*
	(2) medium	5.27 ± 4.51	3.58 - 6.95						
	(3) low	13.06 ± 5.28	11.13 - 15.00						
Age (years)	(1) high	25.10 ± 5.80	23.45 - 26.75	2.25	<0.001*	0.04	0.003*	0.007*	0.001*
	(2) medium	21.00 ± 4.51	19.32 - 22.69						
	(3) low	28.86 ± 5.24	26.94 - 30.78						
Height (cm)	(1) high	173.02 ± 4.91	171.63 - 174.42	4.70	<0.001*	0.08	0.689	0.003*	0.001*
	(2) medium	173.85 ± 4.09	172.32 - 175.38						
	(3) low	169.58 ± 4.06	168.09 - 171.07						
Weight (kg)	(1) high	66.17 ± 4.45	64.90 - 67.44	1.10	0.297	0.02	0.900	0.273	0.542
	(2) medium	65.78 ± 5.19	63.84 - 67.72						
	(3) low	64.52 ± 4.41	62.90 - 66.14						
BMI (kg ∙ m^-2^)	(1) high	22.13 ± 1.35	21.75 - 22.51	0.00	0.208	4.64·10^-3^	0.533	0.621	0.180
	(2) medium	21.78 ± 1.59	21.18 - 22.37						
	(3) low	22.43 ± 1.39	21.92 - 22.94						
%FM (%)	(1) high	11.64 ± 3.53	10.64 - 12.64	0.00	0.328	7.23·10^-4^	0.490	0.879	0.322
	(2) medium	10.79 ± 3.06	9.65 - 11.93						
	(3) low	11.98 ± 2.79	10.96 - 13.01						
FM (kg)	(1) high	7.76 ± 2.60	7.02 - 8.50	0.00	0.644	5.43·10^-5^	0.656	0.900	0.684
	(2) medium	7.28 ± 2.49	6.35 - 8.21						
	(3) low	7.78 ± 2.04	7.03 - 8.53						
LBM (kg)	(1) high	58.41 ± 3.76	57.35 - 59.48	1.67	0.099	0.03	0.900	0.125	0.158
	(2) medium	58.50 ± 3.62	57.15 - 59.85						
	(3) low	56.76 ± 3.58	55.45 - 58.07						
BSA (m^2^)	(1) high	1.79 ± 0.07	1.77 - 1.82	2.84	0.018*	0.05	0.900	0.026*	0.043*
	(2) medium	1.80 ± 0.07	1.77 - 1.82						
	(3) low	1.75 ± 0.07	1.73 - 1.78						

Note: SD - standard deviation, CI - confidence intervals, Experience - sport experience, age - calendar age, height - body height; weight - body weight, BMI - body mass index, BSA - body surface area, %FM - percentage of body fat in total body weight, FM - fat mass, LBM - lean body mass, * - statistically significant difference (p < 0.05).

In the aspect of anaerobic capacity assessed by the Wingate test, motorcycle speedway riders from the medium sport level cluster had a higher total work (kJ) capacity as compared to the low sport level riders (Table 3). However, the difference did not reach a level of significance (p = 0.051).

**Table 3:** Order statistics: mean ± standard deviation and 95% confidence interval with repeated-measures ANOVA and Tukey’s HSD post hoc comparisons of anaerobic performance determined in the Wingate test.

Variables	Clusters: sport level	mean ± SD	95% CI lower - upper	ANOVA			post-hoc comparison p		
F	p	effect size η^**2**^	1 vs 2	1 vs 3	2 vs 3
TW (kJ)	(1) high	17.16 ± 1.35	16.78 - 17.55	1.67	0.044*	0.03	0.900	0.084	0.051
	(2) medium	17.30 ± 1.61	16.70 - 17.90						
	(3) low	16.47 ± 1.27	16.01 - 16.93						
TW/kg (kJ ∙ kg^-1^)	(1) high	259.43 ± 13.87	255.49 - 263.37	0.00	0.330	8.05·10^-4^	0.498	0.871	0.321
	(2) medium	263.35 ± 19.70	256.00 - 270.71						
	(3) low	257.79 ± 11.05	253.73 - 261.84						
PPO (W)	(1) high	733.59 ± 64.64	715.22 - 751.96	2.25	0.072	0.04	0.828	0.061	0.272
	(2) medium	725.36 ± 79.23	695.77 - 754.94						
	(3) low	699.81 ± 44.84	683.37 - 716.26						
PPO/kg (W ∙ kg^-1^)	(1) high	11.08 ± 0.65	10.89 - 11.27	0.55	0.441	0.01	0.900	0.448	0.565
	(2) medium	11.07 ± 0.98	10.70 - 11.43						
	(3) low	10.87 ± 0.69	10.62 - 11.12						
FI (%)	(1) high	24.46 ± 3.82	23.37 - 25.54	1.10	0.116	0.02	0.106	0.459	0.700
	(2) medium	22.44 ± 5.08	20.54 - 24.33						
	(3) low	23.29 ± 4.14	21.77 - 24.81						

Note: SD - standard deviation, CI - confidence intervals, TW - total work, TW/kg - total work per body mass, PPO - peak power output, PPO/kg - peak power output per body mass, FI - fatigue index, * - statistically significant difference (p < 0.05).

Oxygen partial pressure (p = 0.007) and diastolic blood pressure (p = 0.028) were significantly lower among high sports level compared to low sports level cluster (Table 4).

**Table 4:** Order statistics: mean ± standard deviation and 95% confidence interval with repeated-measures ANOVA and Tukey’s HSD post hoc comparisons of gas analysis, cardiorespiratory responses, blood gasometry variables and lactate ions concentrations measured at the Wingate test.

Variables	Clusters: sport level	mean ± SD	95% CI lower - upper	ANOVA			post-hoc comparison p		
				F	p	effect size η2	1 vs 2	1 vs 3	2 vs 3
Hb (g ∙ dL^-1^)	(1) high	14.35 ± 0.91	14.09 - 14.61	2.25	0.060	0.04	0.104	0.134	0.900
	(2) medium	14.82 ± 1.23	14.36 - 15.27						
	(3) low	14.78 ± 0.84	14.47 - 15.09						
[H^+^] (nmol ∙ L^–1^)	(1) high	63.17 ± 5.56	61.59 - 64.75	1.10	0.155	0.02	0.900	0.181	0.234
	(2) medium	63.24 ± 5.88	61.04 - 65.43						
	(3) low	60.95 ± 4.77	59.21 - 62.70						
pCO_2_ (mm Hg)	(1) high	37.94 ± 6.39	36.13 - 39.76	1.10	0.328	0.02	0.900	0.299	0.599
	(2) medium	37.41 ± 4.79	35.62 - 39.20						
	(3) low	36.06 ± 4.58	34.38 - 37.74						
pO_2_ (mm Hg)	(1) high	86.20 ± 12.83	82.56 - 89.85	4.70	0.010*	0.08	0.293	0.007*	0.346
	(2) medium	89.84 ± 7.71	86.96 - 92.72						
	(3) low	93.59 ± 8.23	90.57 - 96.61						
[HCO_3_] (mmol ∙ L^–1^)	(1) high	14.73 ± 1.74	14.24 - 15.23	0.00	0.892	9.05·10^-4^	0.900	0.900	0.880
	(2) medium	14.67 ± 1.76	14.01 - 15.33						
	(3) low	14.87 ± 1.67	14.26 - 15.49						
[La^-^] (mmol ∙ L^–1^)	(1) high	11.59 ± 1.98	11.03 - 12.15	0.00	0.617	2.23·10^-3^	0.229	0.804	0.583
	(2) medium	11.82 ± 1.99	11.08 - 12.56						
	(3) low	11.32 ± 1.98	10.60 - 12.04						
VE_peak_ (l ∙ min^–1^)	(1) high	118.98 ± 13.09	112.91 - 125.05	0.55	0.605	0.01	0.900	0.572	0.802
	(2) medium	117.23 ± 14.47	108.72 -125.74						
	(3) low	113.68 ± 16.26	104.15 - 123.21						
VO_2peak_ (l ∙ min^–1^)	(1) high	2737.54 ± 301.13	2661.06 - 2814.02	0.00	0.944	2.77·10^-4^	0.900	0.900	0.900
	(2) medium	2750.43 ± 339.51	2629.63 - 2871.24						
	(3) low	2721.84 ± 389.22	2568.97 - 2874.70						
VCO_2peak_ (l ∙ min^–1^)	(1) high	3663.72 ± 403.01	3529.00- 3798.44	1.67	0.198	0.03	0.704	0.171	0.618
	(2) medium	3565.90 ± 440.17	3322.76- 3809.04						
	(3) low	3436.26 ± 491.38	3231.65- 3640.87						
HR_peak_ (beats ∙ min^–1^)	(1) high	178.78 ± 19.67	174.72- 182.84	0.00	0.516	4.21·10^-4^	0.520	0.900	0.618
	(2) medium	182.17 ± 22.49	178.23 - 186.10						
	(3) low	179.00 ± 25.60	173.72- 184.28						
SP (mmHg)	(1) high	124.02 ± 13.64	120.34- 127.70	0.55	0.346	0.01	0.900	0.396	0.416
	(2) medium	123.70 ± 15.27	119.26 - 128.14						
	(3) low	127.58 ± 18.24	123.88 - 131.28						
DP (mmHg)	(1) high	74.22 ± 8.16	71.33 - 77.11	2.84	0.029*	0.05	0.900	0.028*	0.113
	(2) medium	75.03 ± 9.26	71.32 - 78.75						
	(3) low	80.26 ± 11.48	76.52 - 84.00						

Note: SD - standard deviation, CI - confidence intervals, Hb - hemoglobin level, [H^+^] - hydrogen ions concentration, pCO_2_ - carbon dioxide partial pressure, pO_2_ - oxygen partial pressure, [HCO_3_^-^] - bicarbonate ions concentration, [La^-^] - lactate ions concentration, VE_peak_ - peak minute ventilation, VO_2peak_ - peak oxygen uptake, VCO_2peak_ - peak carbon dioxide exertion, HR_peak_ - peak heart rate, SP - systolic blood pressure, DP - diastolic blood pressure, * - statistically significant difference (p < 0.05).

### Correlations with sport-level

Statistically significant correlations between the conceptual ISL and measured independent variables considering all participants (without division into clusters) have been found in sports experience (years) (r = 0.45, p < 0.001), age (years) (r = 0.45, p < 0.001), body height (cm) (r = -0.35, p < 0.001), body surface area (m^2^) (r = -0.24, p = 0.009), total work (kJ) (r = -0.25, p = 0.005), diastolic blood pressure (mmHg) (r = 0.24, p = 0.015). No significant correlations were observed with division into clusters. The precise outcomes of all correlations are described in Table 5.

**Table 5:** Results of the Pearson correlation (r) between ‘Indicator of Sport Level’ and the physical parameters (without division into clusters).

‘Indicator of Sport Level’ (N^o^)								
Anthropometrics variables	r	p	Cardiorespiratory and blood gasometry variables	r	p	Anaerobic performance variables	r	p
Experience (years)	0.45	<0.001*	Hb (g ∙ dL^-1^)	0.004	0.958	TW (kJ)	-0.25	0.005*
Age (years)	0.45	<0.001*	[H^+^] (nmol ∙ L^–1^)	-0.14	0.174	TW/kg (kJ ∙ kg^-1^)	-0.18	0.050
Height (cm)	-0.35	<0.001*	pCO_2_ (mm Hg)	-0.06	0.557	PPO (W)	-0.16	0.074
Weight (kg)	-0.11	0.215	pO_2_ (mm Hg)	0.09	0.530	PPO/kg (W ∙ kg^-1^)	-0.11	0.255
BMI (kg ∙ m^-2^)	0.17	0.093	[HCO_3_] (mmol ∙ L^–1^)	0.05	0.624	FI (%)	0.10	0.236
%FM (%)	0.12	0.232	[La^-^] (mmol ∙ L^–1^)	-0.04	0.629			
FM (kg)	0.06	0.591	VE_peak_ (l ∙ min^–1^)	-0.06	0.501			
LBM (kg)	-0.17	0.058	VO_2peak_ (l ∙ min^–1^)	-0.08	0.381			
BSA (m^2^)	-0.24	0.009*	VCO_2peak_ (l ∙ min^–1^)	-0.08	0.353			
			HR_peak_ (beats ∙ min^–1^)	-0.01	0.898			
			SP (mmHg)	0.14	0.132			
			DP (mmHg)	0.24	0.015*			

Note: r - Pearson correlation coefficient, p - calculated probability, * - statistically significant correlation (p < 0.05).

### A mathematical prediction of sport-level

An overall significance of the regression was found (F_(5,105)_=10.35, p < 0.001). The R^2^ was 0.28 (adjusted R^2^ = 0.25), indicating that variables included in the model explained approximately 28% of the variance in sport level expressed by a response variable ‘Indicator of Sport Level’ (ISL). The predictor variables (independent) selected by the OLS method are presented in Table 6 with two significant parameters (body height, p = 0.043, and experience, p < 0.001), and two insignificant parameters in the model (diastolic blood pressure, total work, p > 0.05). The rest of the variables were rejected at the stepwise forward selection stage.

**Table 6:** OLS regression results: predictor variables of sport level (‘Indicator of Sport Level’).

Covariate	Estimate	Standard error	β	t	p	95% CI lower - upper
DP (mmHg)	0.01	0.008	0.11	1.75	0.082	-0.002 - 0.03
TW (kJ)	-0.05	0.06	-0.07	-0.77	0.438	-0.17 - 0.07
Height (cm)	-0.04	0.02	-0.20	-2.05	0.043*	-0.08 - 0.001
Experience (years)	0.05	0.01	0.31	3.93	<0.001*	0.02 - 0.08

Note: Covariate - independent variables, Estimate - regression coefficient, Standard error – standard error of the regression model, β - standardized regression coefficient, t - test statistic. CI - confidence intervals. DP - diastolic blood pressure, TW - total work, height - body height, experience - sport experience. A negative sign of regression coefficient indicates that as the predictor variable increases. the response variable decreases. A positive sign indicates that as the predictor variable increases. the response variable also increases. * - statistically significant (p < 0.05).

The prediction equations for ISL is as follows (1):


ISL = 0.014 ⋅ DP − 0.050 ⋅ TW − 0.041 ⋅ height + 0.056 + 8.999 (1)

Confidence intervals indicated that we can be 95% certain that the slope to predict ISL from DP is between -0.002 and 0.03, TW (-0.17 and 0.07), height (-0.08 and -0.001), and experience (0.02 and 0.08). The Durbin-Watson test indicated a score of DW = 1.89, indicating a score close to zero autocorrelation means no autocorrelation [31]. VIF and TOL values for predictor variables in the model (respectively: 1.06–1.43, 0.69–0.94) showed there is no multicollinearity [38]. The impact of both variables on ISL as reflected by the β-coefficient was as follows: height (β = -0.20), and experience (β = 0.31), highlighting the weak impact of these parameters on ISL.

**Fig 1:**
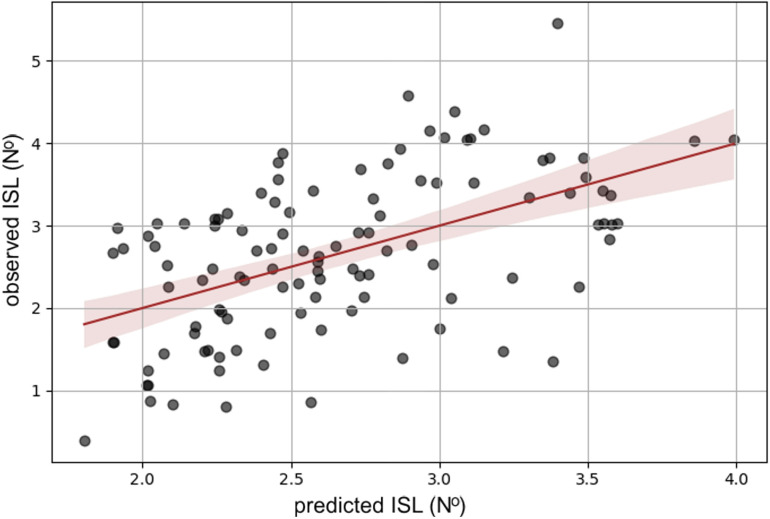
The plot of the regression analysis of observed ISL against predicted ISL. Note: The central red line represents the trend, and the shadow around it represents 95% confidence intervals.

Fig 1 shows a comparison analysis of observed and predicted ISL by regressing one against the other. As expected by the limited R^2^, the data were scattered. The model’s RMSE was 0.81 (N^o^). Predicted ISL equals 2.66 (N^o^), which was approximately 100% of the observed values. The difference between predicted and observed ISL is at the level of <0.001 (N^o^), which means the model predicts the actual values well.

**Fig 2:**
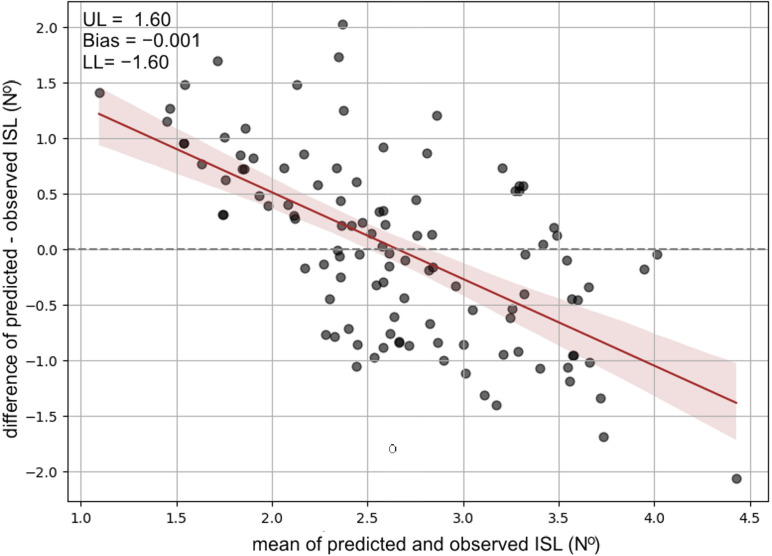
Bland-Altman plots of the prediction accuracy of derived model. Note: UL: upper limit of agreement, LL: lower limit of agreement. The central red line represents the trend. Upper edge of shadow represents upper limit of agreement, and lower edge of shadow represents lower limit of agreement. Area between edges represents model’s accuracy.

In Fig 2, Bland-Altman plots have been presented to evaluate the agreement between observed and predicted ISL. The ISL was accurately predicted with minimal systematic error. The bias was −0.001 (N^o^), indicating no tendency to under- or overestimate the values. The limits of agreement ranged from −1.60 to 1.60 (N^o^), confirming consistency between predicted and observed ISL scores across the whole population.

## Discussion

The primary objective of the present study was to analyze the physical profile of elite male motorcycle speedway riders and to develop a mathematical model to predict the sport level of riders. This study implemented a framework which firstly used data clustering by sport level to compare groups of riders and then created a new sports level indicator to mathematically identify important physical features of athletes that significantly impact sports level. The main findings of this investigation report the optimal range of physical attributes of the best motorcycle speedway riders with significant body height, sports experience, body surface area, and age. Additionally, the findings point out that body height and sport experience were the most important variables for predicting the sport level of riders. The small number of published papers on motorcycle speedway racing and different approaches in assessing the motorcycle speedway riders means that it was not possible to compare, in full, the results of this study with the results of previous studies.

The elite male motorcycle speedway athletes were characterized by average age of 25.10 ± 5.80 years, which made them significantly older than the medium sport level cluster (21.00 ± 4.51 years, p = 0.003) and younger than low sports level cluster (28.86 ± 5.24 years, p = 0.007). Similarly, the elite experience was 9.16 ± 5.62 years and was significantly higher than medium level cluster (5.27 ± 4.51 years, p = 0.004), and lower than low sports level cluster (13.06 ± 5.28 years, p = 0.004). This may indicate the age range for achieving the highest sporting results in this discipline. Martin et. al. [10] highlighted that high performing motorcycle speedway athletes competing in British Speedway Premier League were 25.7 ± 6.7 years with 7.2 ± 5.3 years competitive experience compared with low performer athletes (respectively 23.1 ± 3.3 years, 5.9 ± 3.2 years). Additionally, it represents the role of increasing experience during years of regular training. For example, De Larochelambert et al. [39] reported that peak performance in French alpine skiing athletes was 24.8 ± 0.2 years and was similar to other summer sports. This is consistent with our results. Training process demands a relatively long period of regular training (10–15 years) to reach elite and super-elite level [40]. In turn, some data suggested 7.5 years as an average time from novice to senior national representation in skeleton [41]. Further longitudinal studies could create the potential individual curves to estimate the age at peak performance and progression of elite speedway riders.

When comparing the anthropometric measures, the elite sport level riders (173.02 ± 4.91 cm, p = 0.003) and medium cluster (173.85 ± 4.09 cm, p = 0.001) were noted to be significantly taller than low sport level riders (169.58 ± 4.06 cm). This result corresponds to the body surface area, which is significantly lower for the low sport level cluster compared to the highest level (p = 0.026). Similarly, Martin et. al. [10] reported that high performers motorcycle speedway riders were on the average taller (174.4 ± 4.0 cm) and heavier (71.1 ± 5.7 kg) than low performer riders (respectively 170.0 ± 8.0 cm, 68.4 ± 9.3 kg), however, without significant difference. In fact, this is also confirmed by anthropometric measurements of former elite motorcycle speedway riders, including Rickardsson T. (body height: 176 cm, body weight: 68 kg), Zmarzlik B. (body height: 177 cm, body weight: 69 kg), Hancock G. (body height: 172 cm, body weight: 63 kg) who won from 2005 to 2024 six, five and four Senior World Champion titles, respectively. In general, motorcycle riders in different disciplines, e.g., motocross [42], enduro [43], are relatively light in body weight and short in stature, and riders’ size has been considered influential on sport performance [44]. Other research indicates that in road motorcycle circuit racing (MotoGP), a short stature (166.9 ± 6.7 cm) and low body weight (54.6 ± 6.9 kg) is favorable for optimal race speed, however sample size were adolescent riders [45]. Being overweight is a handicap regarding performance in motorcycling [43].

Several factors may explain these results. The lower body weight of the rider with a constant motorcycle mass results in a lower value of inertia force and higher acceleration of the motorcycle, as well as lower centrifugal force for easier and faster cornering [46]. Optimal weight distribution on the motorbike is very important in the control of the motorcycle [47]. The final mass of the rider and motorcycle affects the engine power-to-weight ratio and consequently the ability to obtain high speed [48]. Since a speedway motorcycle has a minimum weight of 77 kg with an engine capacity equal to 500 cm^3^, a lighter and smaller rider is theoretically advantageous to obtain higher acceleration. In this study, it is important to point out that the weight of all the motorcycles was the same and there was no difference in the riders’ weight (p > 0.05). On the other hand, when the rider is too small, it is necessary to adapt the motorcycle to allow optimum riding [45]. A low weight may compromise the grip on the exit of the curves. Hence, common motorcycle speedway rider’s practice is deflating air from the rear wheel to obtain the appropriate friction force between the tire and the track. On the other hand, the heavier rider requires more muscular force for optimal control of his motorcycle [43].

The height of the rider also has an influence on performance. An excessive height increases the front area of the rider and therefore the aerodynamic drag force increases, resulting in a decrease in speed [45]. The drag force is also proportional to the square of the body’s speed [46]. The current research indicates a speedway motorcycle is designed for high speeds with a low center of gravity (CG), leading to improved precise handling qualities [19]. Lowering the CG often involves better weight distribution and helps in maintaining traction on both tires during cornering, thus enhancing grip [49]. When the rider is on the bike, his and the bike’s CG will combine into a resulting CG. In turn, increasing the height of the rider moves the center of mass upward [50] leading to a decrease in maintaining stability on the bike. From that point of view, a shorter rider might be an advantage.

Hence, motorcycle speedway riders look for a balance between their height, body weight, and muscular strength, and fit the motorcycle to their somatic build. In other categories, such as road races, being small and light does not highly influence performance. Filaire et al. [51] noticed that high level road motorcycling riders were relatively tall (178.4 cm) with an average body weight of 76.4 kg. Similarly, Robertson and Minter [52], reported that road motorcyclists were taller than the general population. The results of these studies can also be related to the cohort of recreational street motorcyclists with average body height of 180.35 cm and average body weight of 78.15 kg [53]. Variations in anthropometry of some of these riders may be explained by intrarace demands placed on them, e.g., covering long and short distances on the track. In other words, bike categories (road, off-road, motorcycle speedway racing) impacted their different physical and/or anthropometric profile.

In addition, mechanical anaerobic performance obtained in the WAnT resulted in TW of medium sport level riders to be higher than low sport level riders (17.16 ± 1.35 kJ, 16.47 ± 1.27 kJ respectively) with a borderline value of p = 0.051. However, this should be interpreted with caution due to the non-significant result of the difference test. Nevertheless, these average value results may have been due to a difference of several years in the riders’ training seniority and adaptation to anaerobic effort. The timing and nature of muscle work during a roughly 60-second motorcycle speedway race (after completing four laps at maximum intensity) indicates lactate concentrations of >12 mmol∙L^–1^ [11] meaning that energy is provided by the combined interaction of aerobic and anaerobic sources of ATP resynthesis [54]. This is also confirmed by Martin et al. [9] who reported that motorcycle speedway athletes achieved high acute responses (%HR_max_) during the race. The number of heats ridden and the rider’s seniority might provide a stimulus to increase anaerobic performance. However, pre- and post- competitive season testing revealed no significant difference in this regard [12]. Similarly, the lack of difference in anaerobic capacity between junior and senior riders was found in the study of Michalik et al. [11]. The inconsistency in the research results indicates that further field analyses of the physiological response of the body induced by a 60-second competition on the track are needed. Also, assessments of relationships between field performance and outcomes of laboratory tests should provide valuable insight in this area. In this case, another approach to anaerobic performance measurement should be implemented such as it was previously reported by Michalik et al. [55] to determine optimal load to achieve real peak power output during the WAnT, because 7.5% of body weight leads to lower values.

Among elite athletes, the analysis revealed the lowest values of oxygen partial pressure (pO_2_) (p = 0.007), and diastolic blood pressure (DP) (p = 0.028) as compared to the low sport level riders. It is noteworthy that both are in the appropriate physiological range, but two aspects can account for these results. Smaller level of blood pO_2_ may be reflected by decreasing ventilation and a slower breathing pattern [56], which lead to reduced metabolic cost of respiratory muscles. Although not statistically significant, the lower value of pO_2_ was potentially because they engaged in a trend to greater total work in maximal exercise during the WAnT, for high (733.59 W), medium (725.36 W), and low (699.81 W) sport level. Moreover, among elite riders, a larger network of blood vessels could result in greater muscle blood supply and lower DP [57]. These results correspond to the studies by Michalik et al. [11] where pO_2_ measured after the Wingate test correlated with heats sum (r = 0.44, p < 0.05) and points sum + bonus points. Therefore, this should be considered in the context of the determinants of sport results. Interestingly, sport level does not differentiate acute cardiorespiratory responses. No other significant changes were observed in anthropometry and physiological parameters that could distinguish the clusters of athletes.

This study demonstrated that only two factors can potentially explain differences in motorcycle speedway racing performance. The strongest predictive variables of sport level in the regression model (R^2^ = 0.28) were body height (p = 0.043) and sport experience (p < 0.001). The prediction model was validated according to the methodology of predictioN mOdels fOr enDurance athLetEs (NOODLE) presented by Kasiak et al. [58]. Comparison analysis (observed vs predicted ISL) with differences of <0.001 (N^o^), the model’s RMSE equal 0.81 (N^o^), and the Bland-Altman plots with bias was −0.001 (N^o^), confirming a well-fitting of the model. However, the weak impact of both variables on ISL has been observed (height β = -0.20, and experience β = 0.31). Even though the model also included diastolic blood pressure, and total work, these variables were not statistically significant. All other analyzed parameters have been excluded by the stepwise forward selection method. The strength of the relationship between model and the dependent variable (ISL) was at the 28% variation of sport level, which meant that the regression model did not explain most of the factors influencing performance of elite athletes. A possible explanation for these results may be that motorcycle speedway racing is a discipline in which a sport’s results are largely determined by other factors including mechanical preparation of motorcycle parts and fitting of the engine to a given motor sports track surface. Also, riders’ specific skills should be mentioned such as time reaction and acceleration phase, which was reported in the previous paper [6], and technical factors related to riding the bike. This kind of motorcycle racing belongs to the group of particularly extreme motorsports in the world [59], so psychological factors should be mentioned as well.

The practical value of this study is indicating key factors determining sport success, which may aid in providing additional support for recommendations for training and could serve as a point of reference in achieving excellence among young athletes. However, the current results only partially allow a direct description of profile characterization of motorcycle speedway riders. That is not to say the current data is not useful. In fact, up to now, this sport has not been studied extensively. This study contributes to the level of knowledge available in the literature related to motorcycle speedway racing and can be an instruction manual for those who want to further study the topic. Moreover, applying the k-means clustering method is nothing new for sports and was used in American football [60], soccer [61], and rugby [62]. A detailed literature review found nothing specific to its application in motorcycle speedway racing for the purposes of identification of sport level groupings. The breakdown by sport level category that was provided is based on the sport level indicators of the riders. Additionally, the parameters of sport level were aggregated and normalized to a single measure, a first in the literature. The resulting new conceptual parameter of ‘Indicator of Sport Level’ (ISL) expressed in dimensionless unit (N^o^) can serve to objectively compare riders on the same scale. The ISL can provide a point of reference of sport level for researchers, coaches and the riders themselves, which was lacking in the literature. For this study, the clustering results show three groups of riders (high, medium, low sport level riders) that is a novel approach in this aspect. These clustering results are not similar to the existing literature about motorcycle speedway racing. In the study by Michalik et al. [11] the grouping factor was the age of the athlete (junior, senior). Furthermore, Martin et al. [10] divided competitors by calculated match average (CMA), unlike the current study’s methodology. Physical profile selection may also be used and generalized to other problems in research or practice, where the objective is to identify important physical features of athletes. Performing cluster analyses at the rider level may be also warranted if practitioners are interested in the similarity between individual athletes on the team.

There are limitations to this study. First, a larger sample size is needed to confirm these findings and allow more objective statistical analysis. Second, examinations should include performing an assessment of body composition using the commonly used dual-energy X-ray absorptiometry (DXA). Using the gold standard measurements in future research would help to more accurately assess anthropometrics among athletes. Third, sport-specific testing protocols assessing anaerobic capacity (Wingate arm crank fitness test), aerobic capacity (maximal oxygen consumption test), upper limb flexor and extensor maximal voluntary contraction (e.g., Biodex system), the functional movement screen (FMS), and simple reaction time test (SRT) should be performed to provide more variables to the prediction model. Fourth, field tests assessing acute and post-exercise cardiorespiratory, biochemical, and perceptual responses should be conducted at maximum intensity training sessions and/or real competitions on the track.

Future research projects can be conducted to overcome the limitations listed above. Additional research could help determine fitness standards required to be competitive at an elite level and may offer a better screening tool for coaches and sport scientists in talent identification processes. Understanding factors such as the design of the motorcycle and the environment in which riders race may also aid in providing additional support to predict the sport level of riders. Furthermore, in order to complete the model and explain other factors influencing the sports performance of elite speedway riders, it is necessary to have more variables. Finally, using more advanced modelling approaches, and intelligent artifacts methods might provide better predictive performance, leading to more precise estimation, more accurate prediction, and finally creating a champ driver physical fitness model.

## Conclusions

In conclusion, this research aimed to find physical predictors of the sport level of the best motorcycle speedway riders. The clusters identified can be used to standardize groups in future research investigating additional variables in motorcycle speedway riders. The created conceptual statistical measure of the sporting level of athletes called ‘Indicator of Sport Level’ (ISL) can be used to objectively compare riders on the same scale. Moreover, the physical profile of elite level motorcycle speedway riders described highlights a greater emphasis on anthropometry measurement characteristics and sport experience rather than physiological parameters. The test of differences between groups of riders revealed significant differences for body height, body surface area, age, and sports experience among elite athletes. This observation was further confirmed by correlation analysis with the sport level. This is also in line with significant body height and sport experience values recorded during the mathematical modeling to predict the sport level of riders. Due to variables in the model explaining only up to 28% of the variance in ISL, it should be used carefully for practice and sports diagnostics. However, it should also be noted that motorcycle speedway racing is a discipline in which, in addition to the human physical and psychological factors, the proper preparation of the motorcycles, including the engine and its components and their adaptation to environmental conditions, e.g., the track surface, which changes during the match, determines the competitor’s success.
